# (*Z*)-3-(4-Fluoro­phen­yl)-1-[4-(methyl­sulfon­yl)phen­yl]-2-tosyl­prop-2-en-1-one

**DOI:** 10.1107/S1600536808034028

**Published:** 2008-10-25

**Authors:** S. Murugavel, P. S. Kannan, A. SubbiahPandi, Ramalingam Murugan, S. SrimanNarayanan

**Affiliations:** a Department of Physics, Thanthai Periyar Government Institute of Technology, Vellore 632 002, India; b Department of Physics, S.M.K. Fomra Institute of Technology, Thaiyur, Chennai 603 103, India; c Department of Physics, Presidency College (Autonomous), Chennai 600 005, India; d Department of Analytical Chemistry, University of Madras, Guindy Campus, Chennai 600 025, India

## Abstract

In the title compound, C_23_H_19_FO_5_S_2_, two of the phenyl ring C atoms and a sulfonyl O atom of the phenyl(methylsulfonyl) group are disordered over two positions with occupancies 0.522 (17):0.478 (17). The methyl­phenyl and fluoro­phenyl rings are essentially planar, with maximum deviations of 0.0059 (8) and 0.0047 (9) Å, respectively. The crystal packing is stabilized by C—H⋯F inter­actions.

## Related literature

For details of the pharmacological activity, see: Turner (2002 ▶); Supuran & Scozzafava (2003 ▶); Masereel *et al.* (2002 ▶); Pellis & West (1968 ▶); Cohen *et al.* (1977 ▶); Csaszar & Morvay (1983 ▶); Lakshmi *et al.* (1985 ▶); El-Maghraby *et al.* (1984 ▶); Dzhurayev *et al.* (1992 ▶); Gewald *et al.* (1966 ▶).
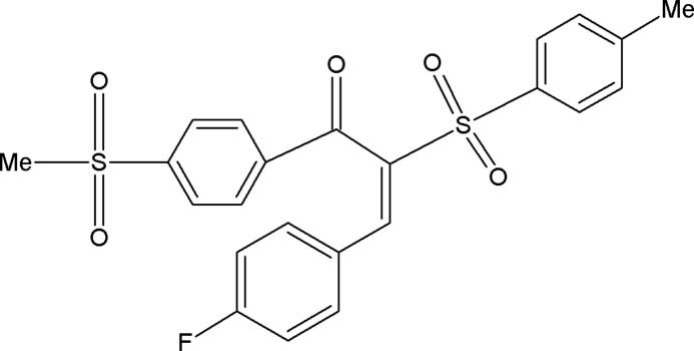


## Experimental

### 

#### Crystal data

C_23_H_19_FO_5_S_2_
*M* *_r_* = 458.52
Monoclinic, 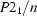

*a* = 9.6962 (7) Å
*b* = 22.8539 (16) Å
*c* = 10.8217 (7) Å
β = 109.672 (2)°
*V* = 2258.1 (3) Å^3^
*Z* = 4
Mo *K*α radiation
μ = 0.28 mm^−1^
*T* = 293 (2) K
0.26 × 0.15 × 0.15 mm

#### Data collection

Bruker APEXII CCD area-detector diffractometer
Absorption correction: none
21889 measured reflections
3994 independent reflections
2543 reflections with *I* > 2σ(*I*)
*R*_int_ = 0.029

#### Refinement

*R*[*F* ^2^ > 2σ(*F* ^2^)] = 0.067
*wR*(*F* ^2^) = 0.241
*S* = 1.01
3994 reflections
293 parameters
4 restraints
H-atom parameters constrained
Δρ_max_ = 0.57 e Å^−3^
Δρ_min_ = −0.25 e Å^−3^

### 

Data collection: *APEX2* (Bruker, 2004 ▶); cell refinement: *APEX2*; data reduction: *SAINT* (Bruker, 2004 ▶); program(s) used to solve structure: *SHELXS97* (Sheldrick, 2008 ▶); program(s) used to refine structure: *SHELXL97* (Sheldrick, 2008 ▶); molecular graphics: *ORTEP-3* (Farrugia, 1997 ▶); software used to prepare material for publication: *SHELXL97* and *PLATON* (Spek, 2003 ▶).

## Supplementary Material

Crystal structure: contains datablocks global, I. DOI: 10.1107/S1600536808034028/fl2219sup1.cif

Structure factors: contains datablocks I. DOI: 10.1107/S1600536808034028/fl2219Isup2.hkl

Additional supplementary materials:  crystallographic information; 3D view; checkCIF report

## Figures and Tables

**Table 1:** Hydrogen-bond geometry (Å, °)

*D*—H⋯*A*	*D*—H	H⋯*A*	*D*⋯*A*	*D*—H⋯*A*
C19—H19⋯F^i^	0.93	2.37	3.274 (8)	167

Symmetry code: (i) 
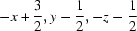
.

**Fig. 1.**
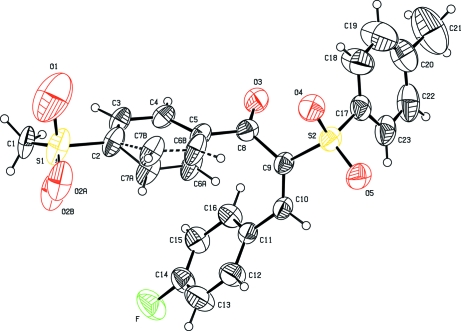
The molecular structure of title compound showing 30% probability displacement ellipsoids. The disordered components are shown.

## supplementary crystallographic information

### Comment

Sulfonamides are an important class of drugs which are known for their pharmacological activities, *e.g.*, anti-microbial and anti-HIV (Turner, 2002), insulin-releasing anti-diabetic, carbonic anhydrase inhibition (Supuran & Scozzafava, 2003), high ceiling diuretic, anti-thyroid and anti-tumor (Masereel *et al.*, 2002). Some sulfur containing Schiff bases [Pellis & West, 1968; Cohen *et al.*, 1977; Csaszar & Morvay,1983; Lakshmi *et al.*, 1985], and their thiophene derivatives [El-Maghraby *et al.*, 1984; Dzhurayev *et al.*, 1992], exhibit  anti-bacterial, anti-cancer, anti-inflammatory and anti-toxic pharmacological activity  [Gewald *et al.*, 1966]. In view of this biological importance, the crystal structure of the title compound has been determined and the results are presented here.

Fig. 1. shows a displacement ellipsoid plot of (I), with the atom numbering scheme. The  F atom  deviates by -0.027 (4)Å from the least-squares of the plane of  ring B. Atoms O2, C6 and C7 are disordered with a ration of 47:52 (Fig 1). The methylphenyl and fluorophenyl rings are essentially planar, with maximum deviations of 0.0059 (8) and 0.0047 (9) Å, respectively.

Atom C19 (*x*, *y*, *z*) donates a proton to the F atom in a neighboring molecule (3/2 - *x*, -1/2 + *y*, -1/2 + *z*) forming a linear chain  along the *a* axis. In addition to Van der Waals interactions, the crystal packing is stabilized by this C–H···F interaction.

### Experimental

1.0 mol of 1-(4-(methylsulfonyl)phenyl)-2-tosylethanone(0.5 g), 1.0 mol of 4-fluorobenzaldehyde(0.18 g) and the sodium hydroxide(0.06 g) was allowed to stir overnight at room temperature to get the title compound. The crude product was filtered and then recrystallized by slow evaporation from chloroform: methanol [9:1] to give the single crystals used for data collection.

### Refinement

The corresponding bond distances involving the disordered atoms were restrained to be equal, and also the same U^ij^ parameters were used for atoms O2A and O2B, C6A and C6B, and C7A and C7B. H atoms were positioned geomentrically (C–H = 0.93Å or 0.97 Å) and were treated as riding on their parent atoms, with *U*_iso_(H)=1.2*U*_eq_(C).

### Crystal data

**Table:**

C_23_H_19_FO_5_S_2_	*F*(000) = 952
*M**_r_* = 458.52	*D*_x_ = 1.349 Mg m^−^^3^
Monoclinic, *P*2_1_/*n*	Mo *K*α radiation, λ = 0.71073 Å
Hall symbol: -P 2yn	Cell parameters from 3994 reflections
*a* = 9.6962 (7) Å	θ = 1.8–25.1°
*b* = 22.8539 (16) Å	µ = 0.28 mm^−^^1^
*c* = 10.8217 (7) Å	*T* = 293 K
β = 109.672 (2)°	Block, colourless
*V* = 2258.1 (3) Å^3^	0.26 × 0.15 × 0.15 mm
*Z* = 4	

### Data collection

**Table:**

Bruker APEXII CCD area-detector diffractometer	2543 reflections with *I* > 2σ(*I*)
Radiation source: fine-focus sealed tube	*R*_int_ = 0.029
graphite	θ_max_ = 25.1°, θ_min_ = 1.8°
ω and φ scans	*h* = −11→10
21889 measured reflections	*k* = −27→27
3994 independent reflections	*l* = −12→12

### Refinement

**Table:**

Refinement on *F*^2^	Primary atom site location: structure-invariant direct methods
Least-squares matrix: full	Secondary atom site location: difference Fourier map
*R*[*F*^2^ > 2σ(*F*^2^)] = 0.067	Hydrogen site location: inferred from neighbouring sites
*wR*(*F*^2^) = 0.241	H-atom parameters constrained
*S* = 1.01	*w* = 1/[σ^2^(*F*_o_^2^) + (0.1393*P*)^2^ + 1.1164*P*] where *P* = (*F*_o_^2^ + 2*F*_c_^2^)/3
3994 reflections	(Δ/σ)_max_ = 0.015
293 parameters	Δρ_max_ = 0.57 e Å^−^^3^
4 restraints	Δρ_min_ = −0.25 e Å^−^^3^

### Special details

**Table:**

Geometry. All e.s.d.'s (except the e.s.d. in the dihedral angle between two l.s. planes) are estimated using the full covariance matrix. The cell e.s.d.'s are taken into account individually in the estimation of e.s.d.'s in distances, angles and torsion angles; correlations between e.s.d.'s in cell parameters are only used when they are defined by crystal symmetry. An approximate (isotropic) treatment of cell e.s.d.'s is used for estimating e.s.d.'s involving l.s. planes.
Refinement. Refinement of *F*^2^ against ALL reflections. The weighted *R*-factor *wR* and goodness of fit *S* are based on *F*^2^, conventional *R*-factors *R* are based on *F*, with *F* set to zero for negative *F*^2^. The threshold expression of *F*^2^ > σ(*F*^2^) is used only for calculating *R*-factors(gt) *etc*. and is not relevant to the choice of reflections for refinement. *R*-factors based on *F*^2^ are statistically about twice as large as those based on *F*, and *R*- factors based on ALL data will be even larger.

### Fractional atomic coordinates and isotropic or equivalent isotropic displacement parameters (Å^2^)

**Table:**

	*x*	*y*	*z*	*U*_iso_*/*U*_eq_	Occ. (<1)
C1	1.3723 (4)	0.4255 (2)	−0.0871 (5)	0.0930 (14)	
H1A	1.3540	0.4621	−0.1335	0.139*	
H1B	1.3282	0.3943	−0.1466	0.139*	
H1C	1.4760	0.4191	−0.0502	0.139*	
C2	1.1672 (4)	0.3762 (2)	−0.0248 (4)	0.0890 (13)	
C3	1.1626 (4)	0.33042 (19)	−0.1075 (4)	0.0806 (11)	
H3	1.2361	0.3260	−0.1441	0.097*	
C4	1.0497 (4)	0.29108 (18)	−0.1363 (4)	0.0769 (11)	
H4	1.0475	0.2598	−0.1918	0.092*	
C5	0.9391 (4)	0.29742 (17)	−0.0836 (3)	0.0679 (10)	
C6A	0.932 (2)	0.3505 (5)	−0.0214 (16)	0.088 (5)	0.522 (17)
H6A	0.8507	0.3582	0.0025	0.105*	0.522 (17)
C6B	0.957 (2)	0.3373 (7)	0.0188 (16)	0.088 (5)	0.478 (17)
H6B	0.8912	0.3383	0.0641	0.105*	0.478 (17)
C7A	1.0415 (11)	0.3915 (9)	0.0058 (15)	0.089 (4)	0.522 (17)
H7A	1.0331	0.4276	0.0425	0.107*	0.522 (17)
C7B	1.0744 (16)	0.3748 (10)	0.0514 (15)	0.089 (4)	0.478 (17)
H7B	1.0927	0.3993	0.1237	0.107*	0.478 (17)
C8	0.8192 (4)	0.25372 (19)	−0.1142 (3)	0.0698 (10)	
C9	0.6772 (4)	0.27111 (17)	−0.0973 (3)	0.0666 (9)	
C10	0.5902 (4)	0.31364 (17)	−0.1618 (4)	0.0702 (10)	
H10	0.5098	0.3228	−0.1370	0.084*	
C11	0.6107 (4)	0.34758 (16)	−0.2697 (4)	0.0669 (9)	
C12	0.5815 (6)	0.4061 (2)	−0.2782 (5)	0.1029 (16)	
H12	0.5487	0.4239	−0.2158	0.123*	
C13	0.5997 (7)	0.4391 (2)	−0.3770 (6)	0.127 (2)	
H13	0.5812	0.4792	−0.3816	0.153*	
C14	0.6457 (6)	0.4119 (2)	−0.4688 (4)	0.0942 (14)	
C15	0.6751 (5)	0.3547 (2)	−0.4645 (4)	0.0861 (12)	
H15	0.7066	0.3373	−0.5280	0.103*	
C16	0.6576 (5)	0.32188 (17)	−0.3637 (4)	0.0773 (11)	
H16	0.6776	0.2820	−0.3592	0.093*	
C17	0.5859 (5)	0.1628 (2)	−0.0386 (4)	0.0824 (12)	
C18	0.6805 (8)	0.1184 (3)	0.0090 (6)	0.132 (2)	
H18	0.7669	0.1254	0.0781	0.159*	
C23	0.4581 (5)	0.1538 (3)	−0.1417 (5)	0.1033 (16)	
H23	0.3927	0.1842	−0.1762	0.124*	
O1	1.4436 (7)	0.3984 (4)	0.1553 (7)	0.261 (4)	
O2A	1.268 (4)	0.4774 (8)	0.071 (3)	0.137 (6)	0.522 (17)
O2B	1.255 (5)	0.4845 (8)	0.021 (3)	0.137 (6)	0.478 (17)
O3	0.8339 (3)	0.20504 (15)	−0.1523 (4)	0.1011 (10)	
O4	0.7600 (3)	0.22936 (15)	0.1392 (3)	0.0991 (10)	
O5	0.5023 (4)	0.25992 (15)	0.0377 (3)	0.1033 (10)	
S1	1.31187 (15)	0.42709 (8)	0.01374 (16)	0.1220 (6)	
S2	0.63094 (12)	0.23274 (5)	0.02559 (10)	0.0801 (4)	
F	0.6602 (4)	0.44442 (14)	−0.5693 (3)	0.1362 (12)	
C22	0.4298 (7)	0.0953 (4)	−0.1942 (6)	0.128 (2)	
H22	0.3440	0.0871	−0.2630	0.154*	
C20	0.5310 (10)	0.0515 (3)	−0.1415 (8)	0.135 (2)	
C19	0.6521 (10)	0.0637 (3)	−0.0419 (8)	0.166 (3)	
H19	0.7192	0.0339	−0.0063	0.199*	
C21	0.4865 (13)	−0.0082 (4)	−0.2075 (9)	0.228 (5)	
H21A	0.5557	−0.0199	−0.2486	0.342*	
H21B	0.3907	−0.0054	−0.2725	0.342*	
H21C	0.4852	−0.0366	−0.1426	0.342*	

### Atomic displacement parameters (Å^2^)

**Table:**

	*U*^11^	*U*^22^	*U*^33^	*U*^12^	*U*^13^	*U*^23^
C1	0.067 (3)	0.122 (3)	0.112 (3)	−0.040 (2)	0.059 (2)	−0.043 (3)
C2	0.060 (2)	0.139 (4)	0.075 (3)	−0.012 (2)	0.032 (2)	−0.029 (2)
C3	0.062 (2)	0.106 (3)	0.086 (3)	0.002 (2)	0.041 (2)	−0.014 (2)
C4	0.068 (2)	0.090 (3)	0.083 (3)	0.011 (2)	0.037 (2)	−0.009 (2)
C5	0.056 (2)	0.098 (3)	0.0552 (19)	0.0054 (19)	0.0253 (16)	−0.0033 (18)
C6A	0.060 (6)	0.161 (7)	0.052 (9)	−0.017 (5)	0.031 (7)	−0.030 (7)
C6B	0.060 (6)	0.161 (7)	0.052 (9)	−0.017 (5)	0.031 (7)	−0.030 (7)
C7A	0.064 (5)	0.143 (11)	0.062 (8)	−0.010 (5)	0.024 (6)	−0.038 (7)
C7B	0.064 (5)	0.143 (11)	0.062 (8)	−0.010 (5)	0.024 (6)	−0.038 (7)
C8	0.071 (2)	0.084 (3)	0.060 (2)	0.010 (2)	0.0291 (18)	0.0063 (18)
C9	0.057 (2)	0.084 (2)	0.062 (2)	0.0006 (18)	0.0245 (16)	0.0043 (17)
C10	0.055 (2)	0.086 (2)	0.074 (2)	0.0004 (18)	0.0274 (17)	0.0023 (19)
C11	0.055 (2)	0.074 (2)	0.073 (2)	−0.0010 (17)	0.0243 (17)	0.0036 (17)
C12	0.130 (4)	0.087 (3)	0.123 (4)	0.028 (3)	0.084 (3)	0.020 (3)
C13	0.186 (6)	0.079 (3)	0.157 (5)	0.030 (3)	0.112 (5)	0.030 (3)
C14	0.112 (4)	0.091 (3)	0.088 (3)	−0.005 (3)	0.044 (3)	0.020 (2)
C15	0.105 (3)	0.090 (3)	0.070 (2)	−0.006 (2)	0.038 (2)	−0.005 (2)
C16	0.096 (3)	0.072 (2)	0.064 (2)	−0.003 (2)	0.028 (2)	−0.0008 (17)
C17	0.079 (3)	0.105 (3)	0.069 (2)	−0.008 (2)	0.032 (2)	0.019 (2)
C18	0.146 (5)	0.101 (4)	0.124 (5)	0.006 (4)	0.012 (4)	0.011 (3)
C23	0.082 (3)	0.150 (5)	0.082 (3)	−0.023 (3)	0.033 (2)	0.021 (3)
O1	0.138 (4)	0.415 (12)	0.189 (6)	−0.047 (6)	−0.001 (4)	−0.062 (7)
O2A	0.118 (7)	0.170 (5)	0.147 (18)	−0.055 (4)	0.076 (14)	−0.082 (7)
O2B	0.118 (7)	0.170 (5)	0.147 (18)	−0.055 (4)	0.076 (14)	−0.082 (7)
O3	0.095 (2)	0.092 (2)	0.133 (3)	0.0088 (17)	0.060 (2)	0.0008 (19)
O4	0.094 (2)	0.130 (3)	0.0670 (18)	0.0013 (18)	0.0190 (15)	0.0136 (16)
O5	0.101 (2)	0.131 (3)	0.102 (2)	0.0228 (19)	0.067 (2)	0.0240 (18)
S1	0.0773 (8)	0.1712 (15)	0.1302 (12)	−0.0381 (8)	0.0517 (8)	−0.0670 (10)
S2	0.0759 (7)	0.1063 (9)	0.0653 (6)	0.0055 (5)	0.0333 (5)	0.0160 (5)
F	0.190 (3)	0.118 (2)	0.125 (2)	−0.002 (2)	0.086 (2)	0.0390 (18)
C22	0.121 (5)	0.187 (7)	0.082 (3)	−0.064 (5)	0.042 (3)	−0.007 (4)
C20	0.170 (7)	0.125 (5)	0.122 (5)	−0.025 (5)	0.066 (5)	0.013 (4)
C19	0.189 (8)	0.121 (6)	0.151 (6)	0.002 (5)	0.008 (6)	0.005 (5)
C21	0.352 (15)	0.160 (7)	0.196 (9)	−0.102 (9)	0.123 (10)	−0.057 (6)

### Geometric parameters (Å, °)

**Table:**

C1—S1	1.402 (4)	C12—C13	1.368 (7)
C1—H1A	0.9600	C12—H12	0.9300
C1—H1B	0.9600	C13—C14	1.369 (7)
C1—H1C	0.9600	C13—H13	0.9300
C2—C3	1.368 (6)	C14—C15	1.336 (6)
C2—C7B	1.410 (6)	C14—F	1.363 (5)
C2—C7A	1.411 (6)	C15—C16	1.380 (5)
C2—S1	1.761 (5)	C15—H15	0.9300
C3—C4	1.369 (6)	C16—H16	0.9300
C3—H3	0.9300	C17—C18	1.350 (7)
C4—C5	1.381 (5)	C17—C23	1.375 (6)
C4—H4	0.9300	C17—S2	1.738 (5)
C5—C6B	1.399 (7)	C18—C19	1.357 (9)
C5—C6A	1.399 (7)	C18—H18	0.9300
C5—C8	1.483 (6)	C23—C22	1.443 (8)
C6A—C7A	1.370 (7)	C23—H23	0.9300
C6A—H6A	0.9300	O1—S1	1.758 (7)
C6B—C7B	1.370 (7)	O2A—S1	1.435 (6)
C6B—H6B	0.9300	O2B—S1	1.435 (6)
C7A—H7A	0.9300	O4—S2	1.431 (3)
C7B—H7B	0.9300	O5—S2	1.438 (3)
C8—O3	1.211 (5)	C22—C20	1.383 (10)
C8—C9	1.503 (5)	C22—H22	0.9300
C9—C10	1.322 (5)	C20—C19	1.329 (10)
C9—S2	1.772 (4)	C20—C21	1.532 (10)
C10—C11	1.469 (5)	C19—H19	0.9300
C10—H10	0.9300	C21—H21A	0.9600
C11—C16	1.377 (5)	C21—H21B	0.9600
C11—C12	1.364 (6)	C21—H21C	0.9600
			
S1—C1—H1A	109.5	C12—C13—H13	120.8
S1—C1—H1B	109.5	C14—C13—H13	120.8
H1A—C1—H1B	109.5	C15—C14—F	118.9 (4)
S1—C1—H1C	109.5	C15—C14—C13	122.5 (4)
H1A—C1—H1C	109.5	F—C14—C13	118.6 (5)
H1B—C1—H1C	109.5	C14—C15—C16	118.6 (4)
C3—C2—C7B	118.5 (10)	C14—C15—H15	120.7
C3—C2—C7A	120.2 (8)	C16—C15—H15	120.7
C3—C2—S1	120.6 (3)	C11—C16—C15	120.7 (4)
C7B—C2—S1	119.4 (9)	C11—C16—H16	119.6
C7A—C2—S1	117.6 (9)	C15—C16—H16	119.6
C2—C3—C4	119.9 (4)	C18—C17—C23	120.5 (5)
C2—C3—H3	120.0	C18—C17—S2	119.5 (4)
C4—C3—H3	120.0	C23—C17—S2	119.9 (4)
C5—C4—C3	120.6 (4)	C17—C18—C19	121.3 (6)
C5—C4—H4	119.7	C17—C18—H18	119.3
C3—C4—H4	119.7	C19—C18—H18	119.3
C4—C5—C6B	119.1 (9)	C17—C23—C22	117.3 (5)
C4—C5—C6A	117.2 (9)	C17—C23—H23	121.3
C4—C5—C8	119.8 (3)	C22—C23—H23	121.3
C6B—C5—C8	119.8 (9)	C1—S1—O2B	109.6 (12)
C6A—C5—C8	122.1 (8)	C1—S1—O2A	128.1 (12)
C7A—C6A—C5	122.7 (16)	C1—S1—O1	107.3 (3)
C7A—C6A—H6A	118.6	O2B—S1—O1	118.3 (14)
C5—C6A—H6A	118.6	O2A—S1—O1	99.0 (12)
C7B—C6B—C5	119.0 (17)	C1—S1—C2	107.9 (2)
C7B—C6B—H6B	120.5	O2B—S1—C2	109.0 (18)
C5—C6B—H6B	120.5	O2A—S1—C2	107.7 (16)
C6A—C7A—C2	116.4 (17)	O1—S1—C2	104.3 (3)
C6A—C7A—H7A	121.8	O5—S2—O4	118.6 (2)
C2—C7A—H7A	121.8	O5—S2—C17	108.9 (2)
C6B—C7B—C2	120.0 (17)	O4—S2—C17	108.9 (2)
C6B—C7B—H7B	120.0	O5—S2—C9	107.52 (18)
C2—C7B—H7B	120.0	O4—S2—C9	107.61 (18)
O3—C8—C5	121.4 (4)	C17—S2—C9	104.35 (18)
O3—C8—C9	120.1 (4)	C20—C22—C23	119.6 (6)
C5—C8—C9	118.4 (3)	C20—C22—H22	120.2
C10—C9—C8	125.3 (3)	C23—C22—H22	120.2
C10—C9—S2	118.9 (3)	C19—C20—C22	119.6 (7)
C8—C9—S2	115.8 (3)	C19—C20—C21	126.6 (9)
C9—C10—C11	125.1 (3)	C22—C20—C21	113.7 (8)
C9—C10—H10	117.4	C20—C19—C18	121.7 (8)
C11—C10—H10	117.4	C20—C19—H19	119.2
C16—C11—C12	118.7 (4)	C18—C19—H19	119.2
C16—C11—C10	121.9 (3)	C20—C21—H21A	109.5
C12—C11—C10	119.4 (4)	C20—C21—H21B	109.5
C13—C12—C11	121.0 (4)	H21A—C21—H21B	109.5
C13—C12—H12	119.5	C20—C21—H21C	109.5
C11—C12—H12	119.5	H21A—C21—H21C	109.5
C12—C13—C14	118.5 (5)	H21B—C21—H21C	109.5
			
C7B—C2—C3—C4	13.9 (11)	F—C14—C15—C16	−178.9 (4)
C7A—C2—C3—C4	−15.3 (11)	C13—C14—C15—C16	0.3 (8)
S1—C2—C3—C4	179.8 (4)	C12—C11—C16—C15	0.2 (6)
C2—C3—C4—C5	0.6 (7)	C10—C11—C16—C15	179.6 (4)
C3—C4—C5—C6B	−12.5 (12)	C14—C15—C16—C11	0.1 (7)
C3—C4—C5—C6A	11.7 (10)	C23—C17—C18—C19	−0.7 (10)
C3—C4—C5—C8	−178.9 (4)	S2—C17—C18—C19	−177.4 (6)
C4—C5—C6A—C7A	−9.9 (17)	C18—C17—C23—C22	0.9 (7)
C6B—C5—C6A—C7A	91 (5)	S2—C17—C23—C22	177.5 (3)
C8—C5—C6A—C7A	−179.1 (10)	C3—C2—S1—C1	23.0 (5)
C4—C5—C6B—C7B	9(2)	C7B—C2—S1—C1	−171.3 (10)
C6A—C5—C6B—C7B	−82 (4)	C7A—C2—S1—C1	−142.3 (9)
C8—C5—C6B—C7B	175.7 (12)	C3—C2—S1—O2B	141.9 (12)
C5—C6A—C7A—C2	−3.9 (18)	C7B—C2—S1—O2B	−52.4 (17)
C3—C2—C7A—C6A	16.6 (15)	C7A—C2—S1—O2B	−23.4 (16)
C7B—C2—C7A—C6A	−77 (4)	C3—C2—S1—O2A	164.5 (12)
S1—C2—C7A—C6A	−178.0 (9)	C7B—C2—S1—O2A	−29.7 (17)
C5—C6B—C7B—C2	5(2)	C7A—C2—S1—O2A	−0.8 (16)
C3—C2—C7B—C6B	−17.0 (19)	C3—C2—S1—O1	−90.9 (5)
C7A—C2—C7B—C6B	84 (3)	C7B—C2—S1—O1	74.9 (11)
S1—C2—C7B—C6B	177.0 (12)	C7A—C2—S1—O1	103.8 (9)
C4—C5—C8—O3	20.9 (6)	C18—C17—S2—O5	−139.9 (4)
C6B—C5—C8—O3	−145.5 (11)	C23—C17—S2—O5	43.5 (4)
C6A—C5—C8—O3	−170.3 (10)	C18—C17—S2—O4	−9.1 (5)
C4—C5—C8—C9	−159.0 (3)	C23—C17—S2—O4	174.2 (3)
C6B—C5—C8—C9	34.6 (12)	C18—C17—S2—C9	105.5 (4)
C6A—C5—C8—C9	9.9 (10)	C23—C17—S2—C9	−71.1 (4)
O3—C8—C9—C10	−118.1 (5)	C10—C9—S2—O5	−3.8 (4)
C5—C8—C9—C10	61.7 (5)	C8—C9—S2—O5	173.3 (3)
O3—C8—C9—S2	65.0 (4)	C10—C9—S2—O4	−132.6 (3)
C5—C8—C9—S2	−115.1 (3)	C8—C9—S2—O4	44.4 (3)
C8—C9—C10—C11	6.0 (6)	C10—C9—S2—C17	111.8 (3)
S2—C9—C10—C11	−177.3 (3)	C8—C9—S2—C17	−71.1 (3)
C9—C10—C11—C16	39.3 (6)	C17—C23—C22—C20	−1.0 (7)
C9—C10—C11—C12	−141.2 (5)	C23—C22—C20—C19	1.0 (10)
C16—C11—C12—C13	−0.7 (8)	C23—C22—C20—C21	−179.7 (5)
C10—C11—C12—C13	179.8 (5)	C22—C20—C19—C18	−0.8 (12)
C11—C12—C13—C14	1.1 (10)	C21—C20—C19—C18	180.0 (8)
C12—C13—C14—C15	−0.8 (9)	C17—C18—C19—C20	0.6 (13)
C12—C13—C14—F	178.3 (5)		

### Hydrogen-bond geometry (Å, °)

**Table:**

*D*—H···*A*	*D*—H	H···*A*	*D*···*A*	*D*—H···*A*
C19—H19···F^i^	0.93	2.37	3.274 (8)	167

Symmetry codes: (i) −*x*+3/2, *y*−1/2, −*z*−1/2.
